# Estimation of renewable energy systems for mobile network based on real measurements using HOMER software in Egypt

**DOI:** 10.1038/s41598-023-43877-2

**Published:** 2023-10-04

**Authors:** Naglaa K. Bahgaat

**Affiliations:** Department of Communications and Electronics Engineering, Faculty of Engineering, Canadian International College (CIC), El Shiekh Zayed, Egypt

**Keywords:** Energy science and technology, Engineering

## Abstract

With the increasing of global awareness of the importance of reducing polluting emissions and maintaining a clean and healthy environment. So, the tendency to generate electric energy from new and renewable sources has become an urgent need to achieve this goal. In this paper an optimal economic cost analysis using hybrid renewable energy sources to generate the electricity needed for long-term evolution mobile phone systems was estimated. The proposed electric system accounts for the reduction of polluting emissions to the environment. The electrical profile of the optimal approaches or the hybrid technology and traditional methods which contain solar photovoltaic’, batteries, wind turbines, diesel generator were estimated and provides the electrical power for a Long-Term Evolution mobile network (LTE) as the load at a remote located area. A real time optimal cost analysis of each proposed network is done based on the real load profile, wind speed and solar radiation was placid on from 6 October city in Egypt. Statistical analysis was performed by varying the systems through comparison to determine the optimal approaches based on the Hybrid Optimization Model for Electrical Renewables software (HOMER).

## Introduction

In recent years, the proliferation of cellular networks, as well as the rapid development of electronic multimedia devices, their applications, and telecom network operators, has induced the deployment of many cellular base stations in remote areas. Simultaneously, United Nations Framework Convention on Climate Change (UNFCCC) established an international environmental treaty to combat “dangerous human interference with the climate system”^1^, partly by stabilizing greenhouse gas concentrations in the atmosphere. The treaty was signed by 154 countries at the United Nations Conference on Environment and Development, informally known as the Earth Summit, held in Rio de Janeiro from June 3–14, 1992. A Bonn-based secretariat was established and implemented in March 1994. The treaty called for continued scientific research, regular meetings, negotiations, and future policy agreements designed to allow ecosystems to adapt naturally to climate change, ensure the safety of food production, and enable the sustainable advancement of economic development^2^. Presently, 174 countries have ratified the Kyoto Protocol to the Convention, which sets out targets and timetables for reducing emissions in industrialized countries.

International efforts to address climate change center around the UNFCCC and its Kyoto Protocol. This treaty represents the international response to compelling evidence, which has been repeatedly collected and confirmed by the Intergovernmental Panel on Climate Change, which climate change is occurring and is largely due to human activities^3^.

Egypt signed the UNFCCC on September 6, 1992, and started implementing it on March 21, 1994^4,5^. The United Nations Climate Conference COP27 was held in Egypt from November 6–18, 2022, in the presence of a majority of world leaders and international organizations, and many agreements were signed supporting the United Nations’ plan to reduce global emissions and temperature. These agreements include:

- Developing dependence on renewable energy sources to generate electric power and implement environmentally friendly projects.
- Financing environmental projects and efforts tackling climate change from major industrial countries or international funds.
- Developing countries to provide the proposed financing mechanisms for the establishment of these environmental projects.

Based on these principles, Egypt has achieved remarkable development in electricity production to match its urban expansion and rapid economic growth. This required a significant increase in electrical energy production, which has risen to between 1500 and 2000 MW annually in the past 5 years^6,7^. With the rapid technological advancement, the significant increase in communication network and the development of mobile technologies, the implementation of 3G and 4G technologies has contributed significantly to an increase in energy consumption in the telecommunications sector, creating an urgent need for electric energy sources to supply these networks with the required energy^8^. Base Stations (BSs) are the main source of power consumption in wireless networks^8,9^, approximately accounting for 57% of total operator energy consumption^10–12^. The global energy consumption of BSs is estimated at about 4.5 GW^13^. Almost more than 50% of the total power consumed by communication networks was found to be consumed by the wireless network access component, while 50%–80% was consumed by the power amplifier. Many BSs that are not connected to the power grid are powered by Diesel Generators throughout the day in rural areas and by a backup power supply for a few hours per day in urban and suburban areas.

Diesel Generators consume a lot of diesel and emit abundant polluting gases, such as carbon dioxide. However, using renewable energy sources instead of DIESEL GENERATORSs reduces operating costs and has various environmental benefits, such as reducing emissions and allowing penetration of cellular networks in Remote Areas (RAs), which ultimately reduces the power system cost. As mentioned before, Egypt has progressed significantly in the last five years regarding electricity production to match its urban expansion and rapid economic growth, where the electricity demand has increased dramatically, between 1500 and 2000 MW annually^14^. Consequently, this urban expansion requires the establishment of communication and mobile networks in areas far from electricity networks^15^. In fact, RAs are usually outside the coverage of government grid electrification.

Thus, to overcome this challenge, “Off-Grid Electrification” (OGE) using Renewable Energy Resources (RERs) are the optimal solution for the electrification of these areas. This is because it may not be economically or technically feasible to rely on traditional grid electrification in these areas^16^.

Conversely, the electric power sector in Egypt has recorded many achievements in the field of renewable energy sources. For example, the production from thermal plants reached 80% in 2022, with a capacity of 162,092 GWh. Moreover, electrical production from renewable energy sources reached 20%, with a capacity of 8663 GWh (wind energy: 12%; solar energy: 2%; hydropower: 6%)^17^. The proposed hybrid system accommodates a mixture of different energy sources (whether renewable generators or power generators used in small grid networks) and is considered the economical solution^18^.

In this study, we propose an optimal system for the hybrid technology to obtain the required electricity for long-term evolution (LTE) networks in RAs according to three factors: geographical location, climatic zone, and actual demand for electricity for loads of the LTE system. Furthermore, we used the Hybrid Optimization Model for Electrical Renewables (HOMER) software as an analytical tool to propose hybrid systems and apply technical and economical between the specifications of the systems used.

The rest of the article is described as follow: Section “Renewable energy resources (RERs)” discusses the Renewable Energy Resources while Section “HOMER software as an optimization tool” presents a brief discussion of HOMER Software as an Optimization Tool. After that the Results and Discussion are presents in Section “Economics overview”. Finally, the Conclusion is illustrated in Section “Case study and system architecture”.

## Renewable energy resources (RERs)

The 2015 United Nations Summit was titled “Transforming Our World: The 2030 Agenda for Sustainable Development”. Among 17 goals were proposed to achieve sustainable development. This agenda includes promoting clean, environmentally friendly, and affordable energy sources (the seventh goal)^19^. Based on their needs, constraints, technological level, and social systems, most countries have focused on RERs to accomplish this goal^20^. Obtaining renewable energy involves generating power from naturally replenished sources instead of conventional sources, such as fossil fuels. RERs can be utilized for transportation, cooling, water heating, and energy generation as given in Ref.^20^.

Renewable energy is mostly obtained in the Middle East and North Africa from the sun and wind. Furthermore, the usage of photovoltaic (PV) cells, concentrated solar power, and wind turbine technologies can produce electrical power depending on the system and location^14^, as given in Table 1 (as adapted from Refs.^21–25^).

**Table 1:** Renewable generation sources.

Systems	Description	Location in Egypt	GP (Watts per unit)
Solar energy	Solar panels or Photovoltaic Technology: convert Global Horizontal Irradiance (GHI) of sunlight into a direct electrical current	Rooftops Isolated central plants	Consists of cells, arrays and modules (One cell produces two watts) Average GP from 250 to 400 watts
	Type: Concentrated solar power (CSP) Technology: by using mirrors Direct Normal Irradiance (DNI) converts into solar thermal energy. Convert the concentrated light to heat then the Electricity is generated	Central plants	About 3 to 5 Gwat
Wind energy	Type: Wind turbines Technology: the blades of the turbine rotated the rotor rotate and producing the Electricity. Wind speed and height affecting on the speed of rotation and the generated power	Rooftops Stand-alone Stand alone	Small turbines: GP from 500 to 1000 watts Small independent turbines: GP up to 2 MW Large independent turbines: generate electricity up to 3 MW or more

Sources depend on Refs.^21–25^.

Notably, OGE does not depend on the electric power generation process, only but needs to contain RERs, and requires also energy storage units (batteries)^26,27^. Table 2 illustrates the classification of OGE according to energy capacity, coverage area, and the number of users^26^.

**Table 2:** Characteristics of the off-grid electrification.

	Mini—grids	Micro-grids	Stand-alone
Capacity of Energy	< 10 MW	< 100 kW	< 20 kW
Coverage area	8–49 km^2^	3–8 km^2^	< 1 km^2^
Number of users	10.000–100.000	1000–10.000	1 to 1000 usually
Size	Communities	Communities	Remote small communities/Individual buildings

### Renewable energy resources in Egypt

**Figure 1:**
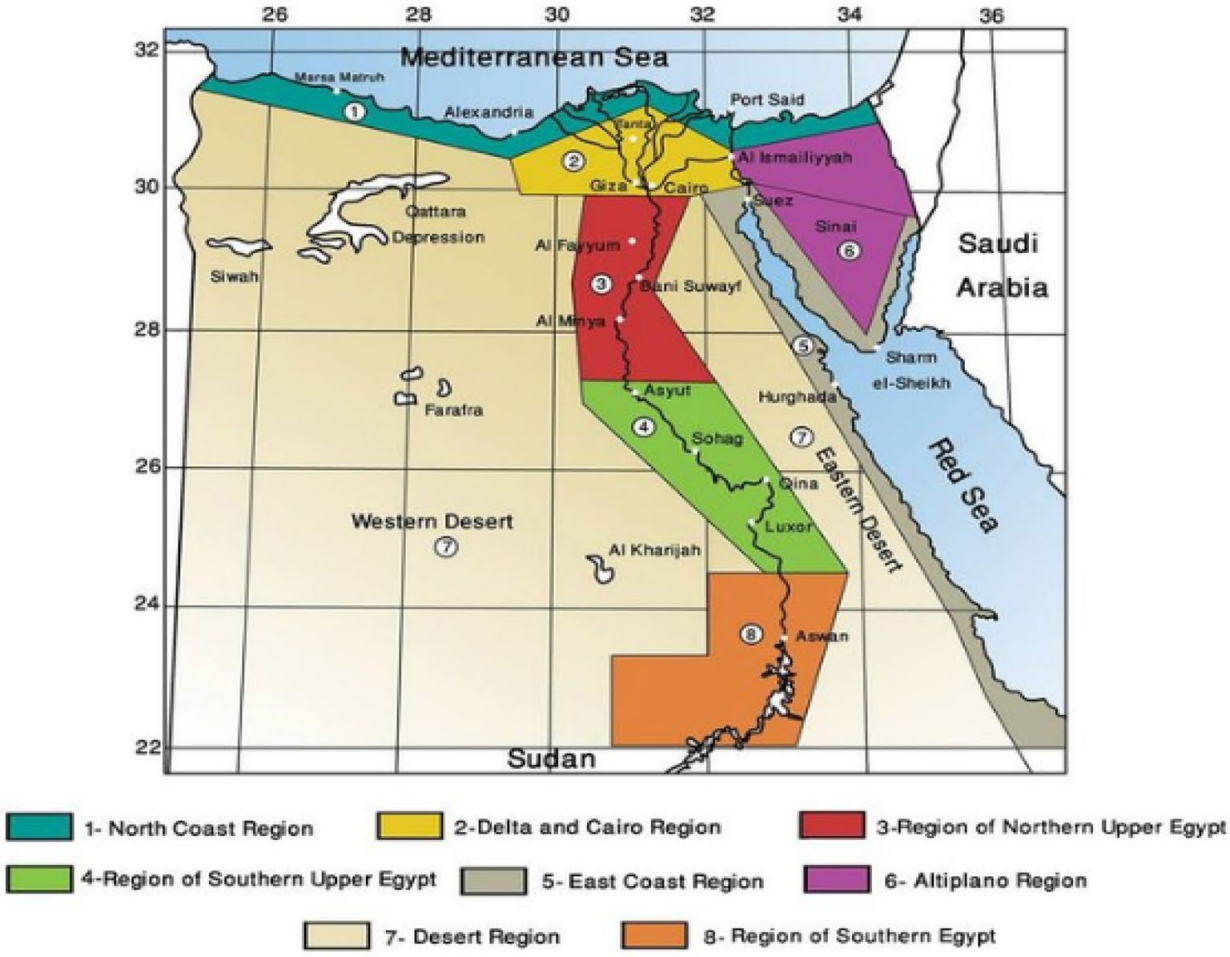
HBRC classification of climatic zone^28–30^.

**Figure 2:**
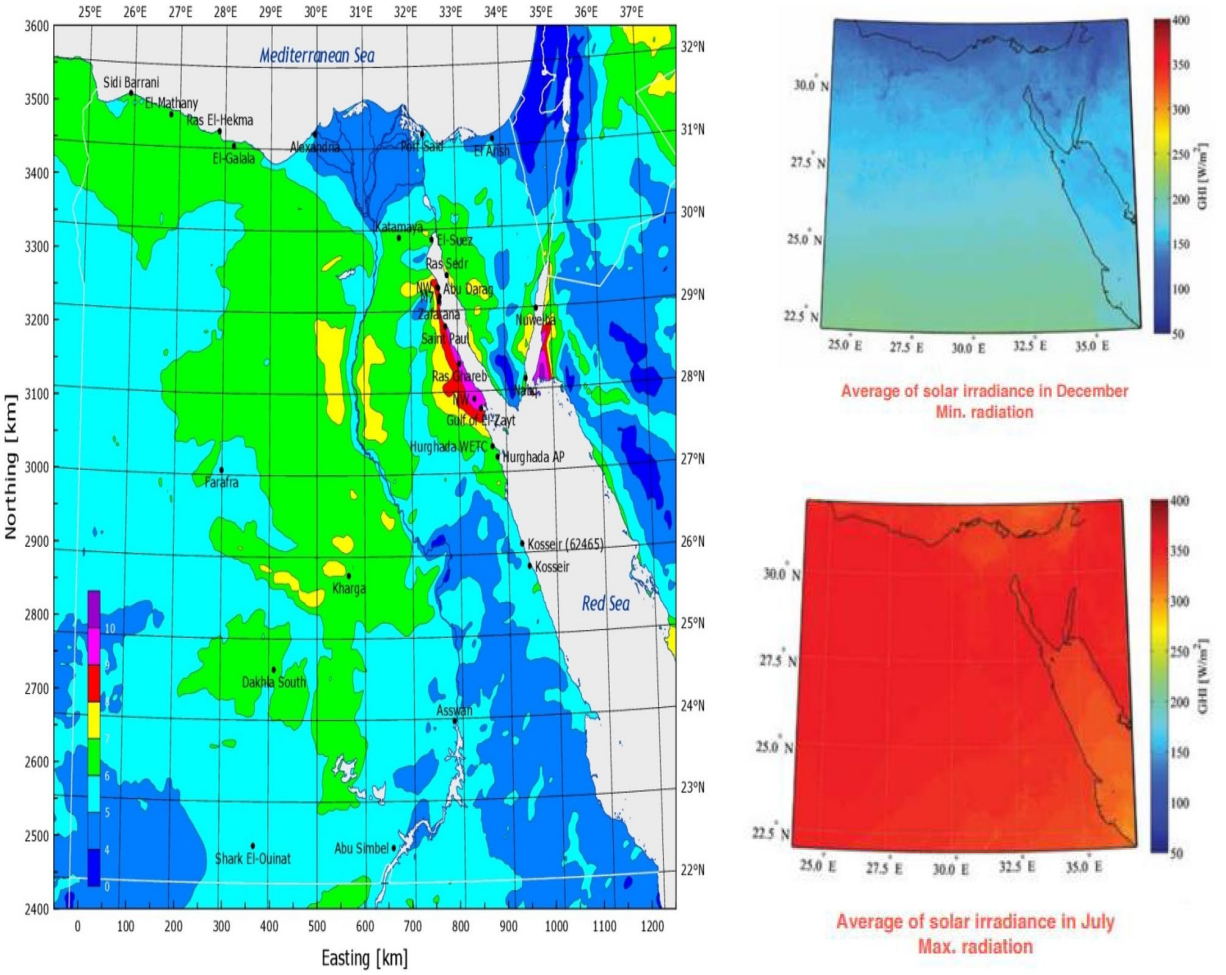
Average wind speed and solar brightness^29,30^.

Consistent with the recommendations of the United Nations in previous climate conferences and COP27, Egypt has been keen to benefit from RERs. Egypt has shown high potential for RERs, as they represented 20% of electricity production sources in the country in 2020^4^. According to the plans of the Egyptian New and Renewable Energy Authority (NREA), the percentage of renewable energy produced is expected to increase to 42% by 2035. In fact, electricity production from RERs is linked to Egypt’s climatic zones. The Housing and Building Research Center divides Egypt into eight different climatic zones Fig. 1^28–30^. Each zone has its unique resources; however, solar and wind energy remain the dominant resources in most zones Fig. 2^29,30^. Several power production projects using RERs have been brought by the NREA^31^, and they have demonstrated their effectiveness and great capabilities^32,33^ as presented in Table 3. These massive projects, whether they are finished or still in the planning stages, are all linked to the country’s electrical grid. However, many off-grid solar energy (PV panel) projects are located in the Western Desert’s Farafra, Abu Minqar, Darb Al-Arbaeen, and Siwa regions, as well as the Eastern Desert’s Marsa Alam, Halayeb, and Shalateen regions^31^.

**Table 3:** Projects of power generation using renewable energy resources.

Off-grid remote areas	Climatic zone	Projects	RER		Capacity
			Solar	Wined	MW
1- North and South Sinai,	East canal zone	Projects under study			
2- Matroh, Salloum	West region				
3- Northern coast, Ras Elhekma	North region				
4- The coasts of the Gulf of Suez and the Red Sea, Nouba, Aswan, Qina, New Valley, Natroun Valley, El-Wahat	South Region	Gebel El-Zeit wind complex		√	580
		Zaafarana wind complex		√	545
		Ras Ghareb		√	260
		West Bakr		√	250
		Gulf Of Suez (under construction)		√	250
		Benban	√		1465
		Plant Kom Ombo	√		26
		Kuraymat solar thermal power-CSP	√		140

### RERs with long-term evolution networks in Egypt

The academic and business fields are interested in the efficient use of energy in wireless networks in accordance with the recommendations of the COP27 summit and concerns about the environment, as well as the demand, cost, and user experience. To enable optimal energy usage in wireless networks and develop computational systems with increased environmental friendliness and energy-saving capability, a unique approach termed “optimizing mobile networks with green energy” has been proposed.

The use worldwide is estimated to be over 3 billion, with each one consuming between 0.2 and 0.4 GW of electricity^33^. Moreover, concerns over the environment, cost, and quality of experience for both service providers and customers have increased.

Recently, some telecom operators have been trying to reduce the power consumption of the equipment they install by 20% yearly. This is in response to the increased power consumption of their central offices, which is due to the increased bandwidth capacity, line card port densities, and demand for intelligent processing, as well as chip implementations with improved intricacy. For telecom companies, an increase in power consumption induces increased operating expenses. Thus, in this study, we determine the optimal method for powering mobile stations and alternatives to conventional energy sources.

The power consumption of telecom sites has been decreased by implementing a solar or wind system design^6,34^ to replace the DG and grid power at the sites. To utilize a PV station as an energy source, it must be considered that the PV units supply electrical energy for loads and batteries given sufficient sunshine exposure. However, PV modules and batteries supply electricity to the loads, given insufficient sunlight exposure. 6 Additionally, the batteries power the loads in the absence of sunlight. Cell towers are built to support one or more cell sites, and they were selected as the site type for the system’s architecture since their radius spanned from 0.5 to 25 miles. Typically, a steel cell tower is constructed. The tower’s base or the leased space contains the transmission equipment of the cell site. The antennas on the tower are connected to the transmission equipment through coaxial or hybrid wires erected in the leased space or positioned at the tower’s base^6^. Cell towers include lattice or self-supporting, guyed, monopoly, and hidden towers which can be designed to resemble trees, signage, light standards, and other structures. Furthermore, to safeguard the equipment from natural or man-made disasters, the equipment shelter should be carefully chosen. To establish a framework that is both secure and safe for sensitive communications equipment. Additionally, the system may experience an energy deficit or disruption due to using a single source of energy.

To operate an off-grid cellular network, integrating renewable energy sources with non-renewable energy sources has been proposed^35,36^, such as integrating a solar PV system with a diesel generator (DG) system. The principal difficulties and optimization solutions of this method are rigorously evaluated by simulating the system with various network topologies using the HOMER optimization software. As a result, a diesel engine has already been fitted by Huawei Technologies to power off-grid cellular networks in Africa and the Middle East, and a hybrid system was developed^37,38^. Although integrating a DG with a solar PV system can solve the source-related issues associated with single renewables, transporting diesel can be challenging in some areas, and burning diesel releases poisonous carbon dioxide. In addition, researchers have advocated for the combined use of several renewable energy sources, such as solar PV systems with wind turbines and solar PV systems with a biomass generator-based supply system^39–42^.

Furthermore, Ericsson has created a wind energy–based hybrid supply system to green-power cellular BSs in off-grid locations after being motivated by the potential of renewable energy^43^. In Germany’s urban areas, Nokia Siemens has also built cellular BSs that are focused on solar PV systems and wind energy^44^. Four types of PV power systems are utilized for cellular BSs^7,45,46^. The first category includes grid-connected systems with no battery backup, which is only used when the utility is operational. Otherwise, the system is turned off until utility power is restored. The second category includes grid-connected systems with battery backup. In these systems, a battery is used to store energy for vital load circuits that must continue functioning during power loss. If there is a daytime power loss, the battery can be supplied with power using a PV array. The third category is the stand-alone system, which is not connected to the grid. Its components include batteries, switches, inverters, and PV arrays, among others.

The batteries that make up PV arrays store the power, which then travels from the batteries to the inverter and BS after being converted from direct current (DC) to alternating current (AC). The fourth category includes stand-alone hybrid systems, which utilize multiple rather than single power sources. There are two kinds of standalone hybrid systems: the first uses diesel, while the second uses petrol^47^. Furthermore, there are two main types of readily available and affordable solar panels on the market: polycrystalline (Poly) and mono crystalline (Mono). The difference between them is that the latter is more expensive than the former per watt but has a superior power density. A typical Poly panel can produce 240 W for $168, whereas a Mono panel of the same size can produce 260 W for $202. Note that the cost of the panels is determined by the watt, and Poly panels cost less than Mono panels per watt^48,49^. The advantages of Mono panels are evident during a large installation of solar panels. For example, the costs of a large installation, shipping, and hardware mounting for Mono panels are observed to be lower per watt, ensuring that the overall cost per watt is comparable to that of a Poly panel installation. This occasionally gives Mono panels a slight cost benefit overall for large projects. However, this advantage is typically negligible in small PV projects.

Both panel types are recommended, although Mono panels often have superior performance for large projects (from 4 to 5 kW in total size) due to their superior energy density per watt. PV systems are connected to numerous arrays made up of modules, and each module is composed of the number of cells. When a panel is rated at 7 its maximum wattage, it can produce this much power per hour of sunlight under direct normal solar insolation (DNI), 1000 watts per meter conditions. Daytime hours are distinct from sun hours. Every region has an equal number of “sun hours”, solar insolation, radiation, or irradiation. These are yearly averages that account for the average amount of rain or cloud cover in the area. The kWh per square meter per day is the unit for measuring solar power. The solar energy required each day must be considered to determine the wattage required. This means the amount of electricity needed to operate the system when the sun is down plus the amount of power to be saved in batteries for the operation of off-grid applications, such as our telecom site, after the sun goes down. Moreover, the size of the battery is based on the number of “evening hours.”

The actual energy required by the system will be reduced at increased temperatures and increased at reduced temperatures. The optimally accurate measurement of real energy at various temperatures where the DC system may need to operate under cloudy or rainy conditions^50^ that affect the normal capacity of the panels to deliver the rated power can be obtained using a string sizing calculator that uses the values on the panel specification label. Additionally, the most effective and efficient method involves the solar storage controllers regulating the energy flowing from the PV array and transferring it directly to the batteries through a DC-coupled scheme or to the load. The ability of the charge controller to supply energy to the batteries as quickly as possible is an important characteristic. The batteries are connected to the solar panels through the charge controller. There are two technological options for a charge controller: pulse width modulation (PWM) or maximum power point tracking (MPPT). The MPPT controllers eliminate the peak energy of the solar panels^51^, although they are more expensive than PWM controllers.

MPPT controllers employ an algorithm based on the maximum power point of the system to manage the voltage of the PV panel and monitor its output. Furthermore, they examine the output of the PV panel and compare it to the battery’s voltage before adjusting the voltage to inject the maximum current into the battery. MPPT controllers can increase charging efficiency by up to 30% due to their unique arrays, which have an input voltage higher than the battery bank, current capacities up to 80 A, and warranties typically longer than those of PWM devices^49^. MPPT is the only method for developing systems and provides considerable flexibility. Additionally, MPPT is the only method for controlling grid connection modules for battery charging, and it provides excellent flexibility for system development as presented in Refs.^51–53^.

There are two types of solar DC cables for connecting design components and wiring solar panels^49,51^. The first type is “module wires” or “string cables,” which are typically embedded into solar PV panels, and the appropriate connectors are used to link them. The positive and negative wires from the string cables are connected to the generator connection box (or directly to the solar power inverter) using the second type of solar DC cable, which is a special extension cable known as the “DC main cable” or “DC primary cable.” Typically, PV cables with cross-sectional areas of 2.5 mm^2^, 4 mm^2^, and 6 mm^2^ are utilized depending on the output power of the modules. DC cables are used for outdoor applications. The DC primary cable is typically used depending on the output power of the module. To prevent an Earth fault and a short circuit, positive and negative wires should not be connected in the same cable. Single-wire cables with double insulation have been established as a useful option with excellent reliability. The batteries make up the third component. Moreover, they are attached to the charging controller for protection from overcharging or discharge; however, the battery life is reduced after reaching the limit in both cases. To regulate the amount that should be charged or discharged, the batteries have a depth of discharge.

### Power storage batteries

A battery is a device that can convert chemical energy into electrical energy. Furthermore, the output voltage and current capacity of a battery are rated. Rechargeable batteries are often available in outputs of 6 V and 12 V. The amount of energy a battery can store determines its capacity. A large battery should be able to store a significant amount of energy, and its current capacity is given in Amber hour (Ah). This indicates the amount of current that can be drawn from a fully charged battery and the speed with which it can be drawn. For example, a 10 Ah battery can deliver 1 A of electricity for 10 h or 10 h at 10 A. The Ah capacity of the battery increases with an increase in battery size. It should be noted that the types of batteries are different according to the storage capacity, materials like Lead Acid (LA), Lithium-Ion (Li-Ion), and Nickel–Iron (Ni–Fe), and economic feasibility of each type, considering also load following (LF) and cycle charging (CC) as discussed in Refs.^54,55^. Which prove that The Ni–Fe battery-based Integrated Hybrid Renewable Energy System (IHRES) with cycle charging (CC) strategy provided a minimum life cycle cost (LCC) and cost of energies (COE). Since the “power” battery type is the most popular in the telecom industry, it should be used in this model NAS battery, which is made of sodium (Na) and sulfur (S). NAS Battery Supports Evening Peak Demand in hybrid PV/Wind station, and solves Over-Generation of PV in Main Grid and it makes the wind power stable & schedulable, more environmental friendly by load following and energy shift as discussed in Ref.^56^. The battery comprises six cells with a total capacity of 150 Ah and 12 V per cell. To obtain the 48 V necessary for the telecom site, four string batteries were connected in a specific order^55^ after classifying each layout element according to its purpose and calculating the loads at the telecom site. The three components of telecom site equipment regarding the on-site lights are transmission, communication, and energy equipment. Furthermore, the PV system can only be constructed for 2G and 3G technologies. The 4G technology consumes a lot of power; thus, integrating it into the site will be impractical. Additionally, the site has only two industries and not three because it was developed in a desert area with a tiny population, eliminating the necessity for a third industry to serve the selected area.

## HOMER software as an optimization tool

**Figure 3:**
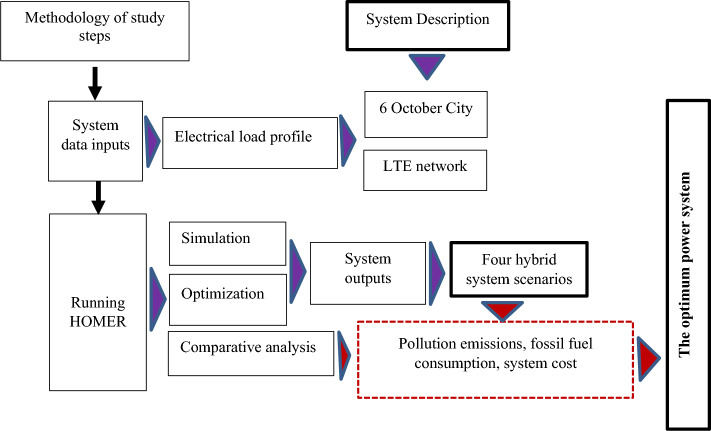
Study Frameworks by HOMER Software.

To enhance the viability of hybrid systems, simulation analysis software, HOMER, is employed. It is accepted worldwide for maximizing micro grid design across all industries. Moreover, over 250,000 system designers and developers in more than 190 countries have used it^33^. It was originally created by the National Renewable Energy Laboratory to serve as a design and investment selection tool for picking the optimal arrangement, size, location, and dispatch of the many energy sources supplying the off-grid energy system^32^. The program has three strong capabilities: simulation, optimization, and sensitivity analyses (Fig. 3).

The simulation software tries to model a workable system for all likely equipment combinations considered. Further, it can discover the least expensive solutions for micro grids or other distributed generation electrical power systems since the optimal designs and configurations were acquired through the optimization analysis. Other sensitivity analyses compare hundreds of options to determine the impact of different variables, such as wind speed and fuel prices, and warn how the optimal system changes as a result^32,57^. Numerous pieces of information on renewable energy sources, energy storage technologies, control strategies, and financial constraints are required for HOMER analysis.

## Economics overview

The financial criteria, such as levelized cost of vitality, system net present cost, system capital expense, and salvage cost, can be used to assess the viability of the developed hybrid system. Based on these economic parameters, HOMER simulates and produces the optimal possible outcome.

### Net present cost (NPC)

A critical cost parameter in HOMER programming^11^. The NPC is defined as the sum of all the expenses absorbed by the program over the system's life expectancy, less the value of the money made during the venture's life expectancy. Net present value includes the initial capital cost, replacement cost, operational and maintenance expenses, fuel cost, and so on. The NPC is mathematically expressed by Eq. (1)^58,59^.

1 $$NPC=\frac{Cannual.Tot.}{CRF(i,Lproject)},$$

where: Cannual. Tot is absolute annual cost in $/year, CRF is a capital recovery factor, i is the interest rate, Lproject is designed project life time.

### Levelized cost of energy (LCOE)

As determined by HOMER Levelized cost of energy is the amount of useful power generated by the system. The LCOE is the proportion of the complete yearly expense of power created to the total useful power generated by the system^59^. The LCOE equation is presented in Eq. (2) as Ref.^59^. The LCOE is defined in HOMER as the mean cost/kWh following Eq. (2).

2 $$LCOE={C}_{annual,tot}/\left({E}_{primary ,AC}+{E}_{primary,DC}+{E}_{grid, sales}\right),$$

where: E_primary, AC_ represents the primary load served in kWh/year. E_primary,DC_ represents the primary load serviced in kWh/year. Egrid,sales represents the total amount of energy sold from the grid in kWh/year.

The relationship in Eqs. (3) and (4), where (Cannual,tot) is the overall annual cost in dollars ($/year), (CRF) is the capital recovery factor, (Rproj) is the project lifespan in years, and i is the interest rate percent, is used to determine the total NPC in HOMER^58–60^.

3 $${C}_{NPC}=\frac{{C}_{annal, tot}}{CRF\left(i, {R}_{proj}\right)},$$

4 $$CRF= \frac{i{\left(i+1\right)}^{{R}_{proj}}}{\left[{\left(i+1\right)}^{{R}_{proj}}-1\right]}.$$

### Renewable fraction (RF)

The renewable percent is the total renewable power provided by sustainable power sources divided by the total power produced by the entire system^59^. For this investigation, the RF must be as high as possible in order to reduce the impact of greenhouse emissions, which are ozone-depleting substances discharged by traditional diesel generators, even though this would have an impact on the NPC due to the expense of renewable power sources. It can be written analytically as Eq. (4)^59^.

5 $$\mathrm{RF}=\mathrm{Renewable \,Fraction \,}\left(\mathrm{\%}\right)=1-\frac{\sum {P}_{diesel}}{\sum {P}_{renewable}},$$

where P_diesel_ is the diesel generator's output. P_renewable_ is the output from the renewable resources.

## Case study and system architecture

In this section, an electrical system is proposed to supply the LTE mobile phone station located in the 6th of October City, Egypt, with the electrical energy necessary for its operation. HOMER software to build, simulate, and optimize a PV and wind turbine as the primary (RER) source of power generation in LTE mobile networks in RAs, as described in the framework by HOMER Fig. 3. Several energy sources will be studied, which will be discussed as follows:

### Section one: the electrical load profile for Bss

**Figure 4:**
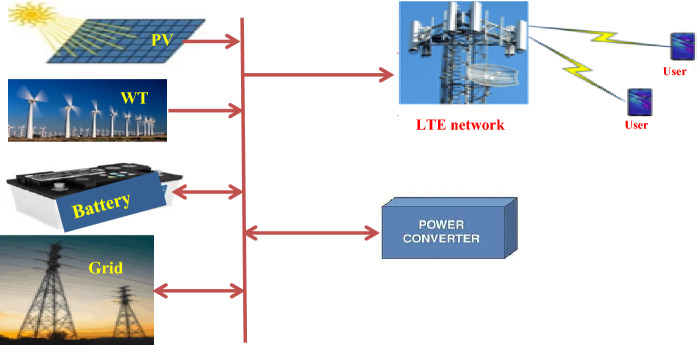
LTE station with different power sources.

The electrical load profiles for the energy needs of the system during the day were studied, and the capacity of the electrical station required to operate the mobile station was estimated. Figure 4 shows the schematics of the proposed hybrid solar PV/wind-powered macro-cellular system^6^. BSs are described as being a DC load, while some AC loads, including AC lamps and air conditioners, are linked to the BSs. A wind turbine and solar PV panels make up the proposed system. A battery bank is connected to the supply system to maintain a continuous power supply and improve system dependability while providing backup power in the event of RES shortages or outages. In addition, a converter is required to switch from AC to DC or vice versa. The mathematical modeling of the main components of the proposed system is presented below:

Table 4 shows an actual predicted load that operates throughout the day and the electricity used for these loads^6^, whereas lighting lambs only operate during periods of darkness and are anticipated to operate from 7 p.m. to 7 a.m. HOMER is used to run calculations to create a practical design for a PV station and determine the batteries to be used. The PV system involves several components that should be selected based on the type of panels, the location of the site, and the apps. The essential components of the solar PV system are the solar charge controller and the battery bank, designed with HOMER.

**Table 4:** Telecom site loads and working hours.

Type	Equipment	Function	Power consumption	Working hours
Telecom	RUS (Radio units)	Send and receive radio signals	370 W	24 h
	DUG&DUW (Digital unit)	Control processing	80 W	24 h
	RRU (Remote radio unit)	Transceiver to the base station	150 W	24 h
	BBU (Baseband unit)	Baseband processing	200 W	24 h
Transmission	RTN 980 (Radio transmission system)	Transport of microwave signals	300 W	24 h
Power	PBU (Power & battery unit)	Power distribution and saving	1400 W	24 h
Light	DC lambs	Lighting	25W	14 R

### Section two: HOMER analysis

The necessary data and load profiles specified in the previous section were used as input for the HOMER software. The following two processes were achieved:

The simulation process was performed using the load profiles of the LTE network in the 6th of October City as presented in Ref.^6^. First, the total power and energy consumption of all loads were determined and Telecom Site Loads were described as shown in Table 5^6,60,61^. Second, the Total Power Requirement of the system loads was determined to multiply the power consumption of each component to be supplied by its working hours so that the energy of the equipment could be obtained. Finally, the total energies of all equipment were added. The total energy requirement of the system (E) = number of units × rating of equipment with DC components as illustrated in Table 6^6^.

**Table 5:** Telecom site loads.

Type	Equipment	Function	Power Consumption	Working Hours
Telecom	Radio units RUS	Send and receive radio signals	370 W	24 h
	Digital unit DUG& DUW	Control processing	80 W	24 h
	Remote radio unit RRU	Transceiver to the base station	150 W	24 h
	Baseband unit BBU	Baseband processing	200 W	24 h
Transmission	Radio transmission system RTN 980	Transport of microwave signals	300 W	24 h
Power consumed	Power & battery unit PBU	Power distribution and saving	1400 W	24 h
Light	DC lamps	Lighting	25W	14 h

**Table 6:** Total electrical power comsumption.

	Load 1	Load 2	Load 3	Load 4	Load 5	Load 6	Load 7	LOAD
	Lighting	Rus	DUG	RRU	BBU	PBU	RTN 980	
Load in W	25	370	80	150	200	1400	300	W
12:00 ~ 1: 00AM	√	√	√	√	√	√	√	2525
1:00 ~ 2: 00 AM	√	√	√	√	√	√	√	2525
2:00 ~ 3: 00 AM	√	√	√	√	√	√	√	2525
3:00 ~ 4: 00 AM	√	√	√	√	√	√	√	2525
4:00 ~ 5: 00 AM	√	√	√	√	√	√	√	2525
5:00 ~ 6: 00 AM	√	√	√	√	√	√	√	2525
6:00 ~ 7: 00 AM	√	√	√	√	√	√	√	2525
7:00 ~ 8:00AM		√	√	√	√	√	√	2500
8:00 ~ 9:00AM		√	√	√	√	√	√	2500
9:00 ~ 10:00AM		√	√	√	√	√	√	2500
10:00 ~ 11:00AM		√	√	√	√	√	√	2500
11:00 ~ 12:00AM		√	√	√	√	√	√	2500
12:00 ~ 1.00PM		√	√	√	√	√	√	2500
1:00 ~ 2:00PM		√	√	√	√	√	√	2500
2:00 ~ 3:00PM		√	√	√	√	√	√	2500
3:00 ~ 4:00PM		√	√	√	√	√	√	2500
4:00 ~ 5:00PM		√	√	√	√	√	√	2500
5:00 ~ 6:00PM	√	√	√	√	√	√	√	2525
6:00 ~ 7:00PM	√	√	√	√	√	√	√	2525
7:00 ~ 8:00PM	√	√	√	√	√	√	√	2525
8:00 ~ 9:00PM	√	√	√	√	√	√	√	2525
9:00 ~ 10:00PM	√	√	√	√	√	√	√	2525
10:00 ~ 11:00PM	√	√	√	√	√	√	√	2525
11:00 ~ 12:00PM	√	√	√	√	√	√	√	2525
Hrs	14	24	24	24	24	24	24	60,350
Energy	350	8880	1920	3600	4800	33,600	7200	

The optimization process was implemented to sort and filter the designed systems following the defined criteria. Four systems were deduced. The first contained the LTE network with only the PV station. The second contained the DIESEL GENERATORS integrated with the PV station panels, the converter to convert the AC power into DC, an energy management system that actively monitors and manages the LTE network loads, and the battery used to store excess energy. The third contained the PV system connected to the grid along with the battery and converter. The final system replaced the PV system with a wind turbine as another renewable energy source.

## Results and discussions

Calculations were conducted for each system using HOMER soft wear. Furthermore, statistical analysis was conducted using the comparison approach to determine the optimal system.

### System containing only the photovoltaic station

**Figure 5:**
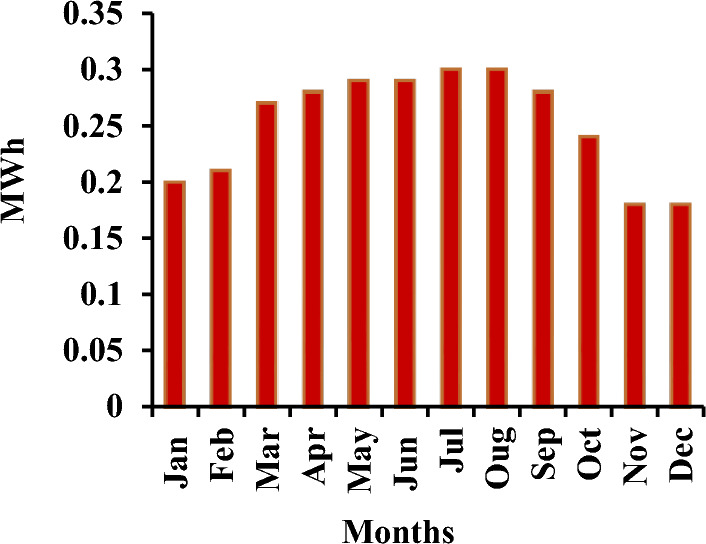
HOMER Schematic for PV system.

**Figure 6:**
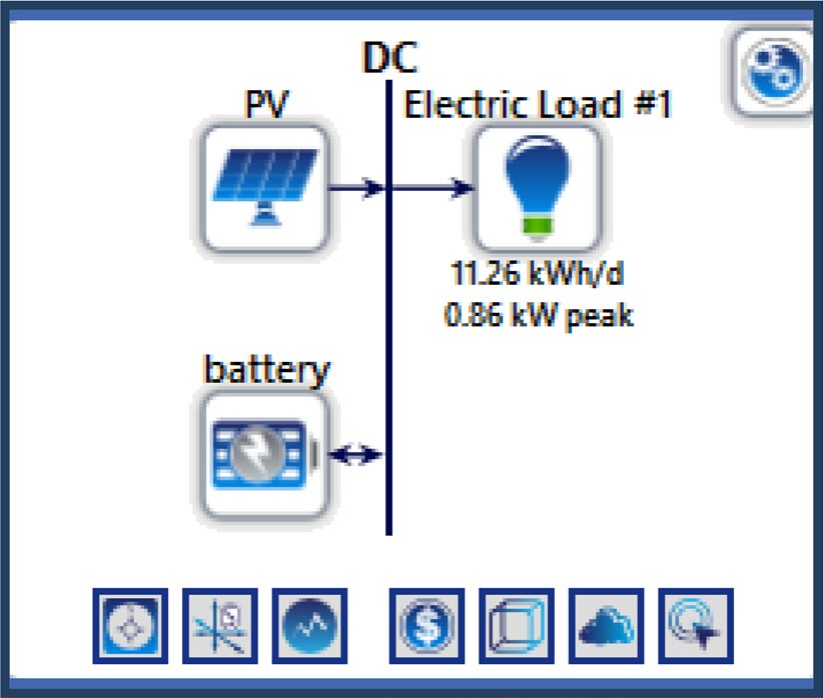
Screenshot for PV monthly electrical production in 6 October city.

**Figure 7:**
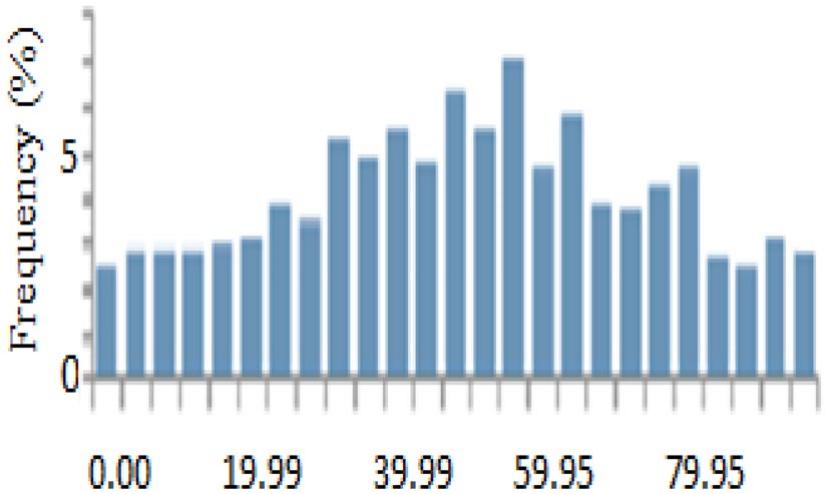
Screenshot for charging capacity of the battery.

**Figure 8:**
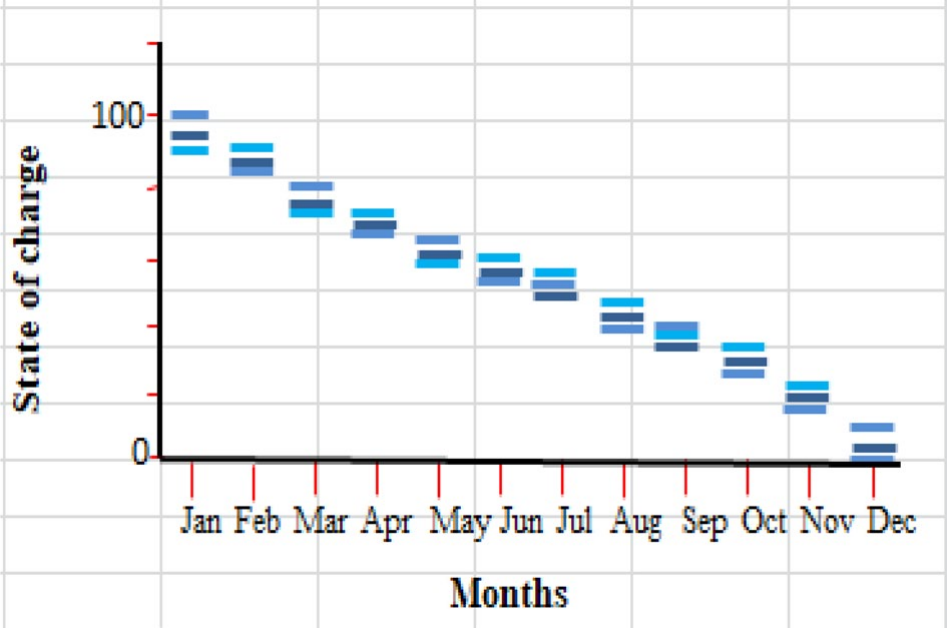
Screenshot for state of battery charge.

The first system for the power supply of BSs was designed as a stand-alone model, which consists of the following features:

- A generic flat plate PV module, which provides 2976 kWh/year as presented in Eq. (1). Given the power requirements for the continuous operation of the mobile station as shows in Tables 4 and 6, the PV system was considered to sufficiently cover the peak electric loads. The PV panel would require systems and have high initial costs. However, this cost will be compensated within a few years, making it a good economic return.
- Energy storage is used to overcome the intermittent nature of renewable energy sources. Excess energy is stored in batteries^6^. Conversely, when there is a high demand for energy or during night periods when there is no sunlight, the batteries are discharged. However, the permissible limit of discharge is observed. Different batteries were observed, and the least expensive and most efficient battery was selected. This was the BASF NAS lithium-ion battery^64,65^, which has a large capacity (1250 kWh), long duration (4.4 h), discharge output (286.1 kW max), and long lifetime (20 y or 6,250,000 kWh). Table A1 (Appendix A) illustrated a comparison of batteries with other studies.
- Inverters are not used because all the system loads are DCs. Thus, the system was low cost^52,61,62^. Figure 5 shows the PV system connected to the LTE network. The monthly electrical production of the panels is illustrated in Fig. 6. While Fig. 7 presents the charging capacity of the battery, moreover Fig. 8 illustrates the state of the battery charge. Notably, this system does not emit any pollutants into the environment because it does not utilize fossil fuels or conventional generators, which require fossil fuels to operate. Thus, this is a significant benefit of the system. Table A2 (Appendix A) presented the system comparison with previous studies^63–65^.

The output values generated by the HOMER algorithm for this system are displayed in Table 7.

**Table 7:** The results using HOMER using PV panels only.

Production	Quantity	Units
PV array (Generic flat plate PV)	2976	kwh/year
NAS battery	1	Unit
Mean output	0.340	kW
Maximum output	164	kW
Mean out per day	815	kWh/d
Hours of operation	4385	hrs/year
Losses	319	kWh/year
Operation cost	501.804	$
Carbon dioxide	0	Kg/year
Carbon monoxide	0	Kg/year
Unburned hydrocarbons	0	Kg/year
Particulate matter	0	Kg/year
Sulfur dioxide	0	Kg/year
Nitrogen oxides	0	Kg/year

### System containing the photovoltaic panel and diesel generator

In this system, a DIESEL GENERATORS was integrated with the PV electrical system to provide another source of electrical energy for the loads at night or when the solar PV system was not in operation. In this case, an inverter must be used to convert power from AC to DC, and a battery must be used to store excess electrical energy.

**Figure 9:**
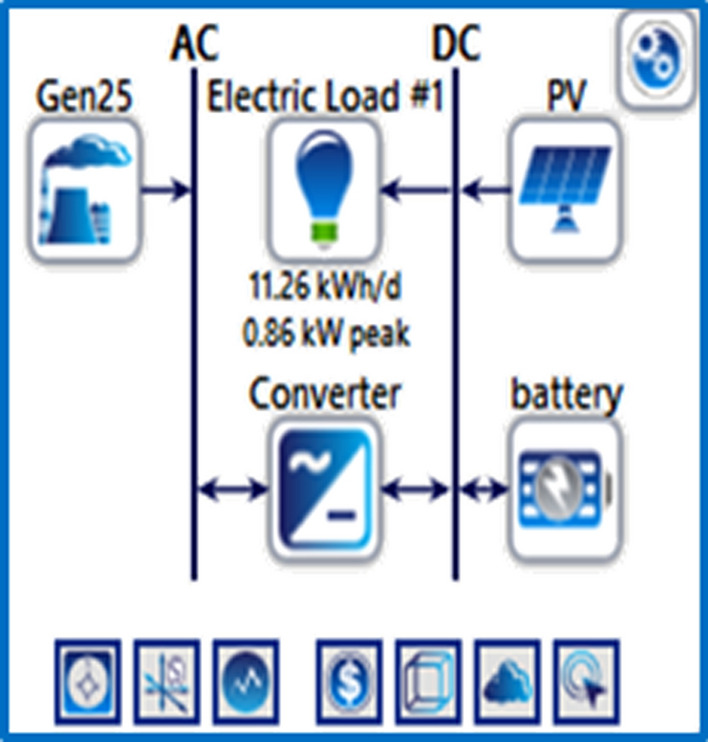
HOMER Schematic for PV and diesel generator.

**Figure 10:**
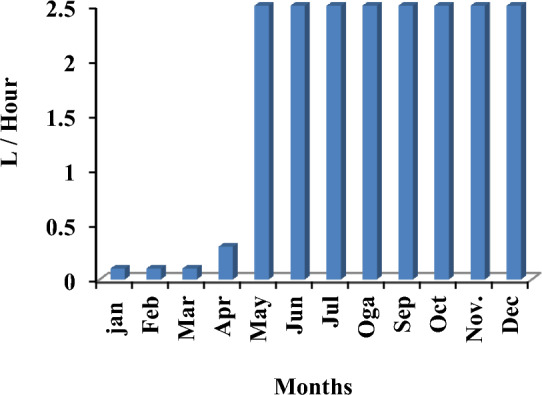
Screenshot for PV Monthly electrical production of the system.

**Figure 11:**
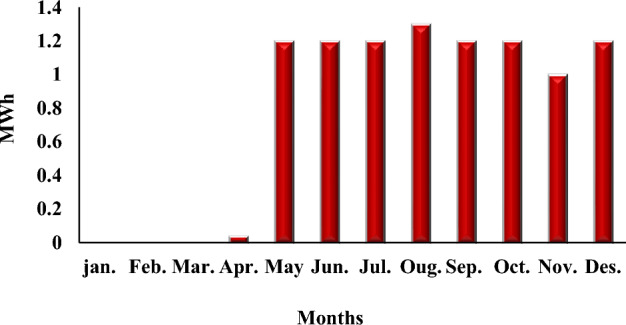
Screenshot for Average fuel consumed per hours.

The system components used are a generic 25 kW fixed capacity DIESEL GENERATOR, a generic flat plate PV module, a NAS battery, and a system converter. Notably, costs will increase to introduce other components absent in the previous PV system. We also note the emergence of gas emissions polluting the environment, which is expected due to the use of fossil fuels to operate the electric generator. Figure 9 presents the system constants, while Fig. 10 shows the monthly electrical production of the PV system. Figure 11 illustrates the average fuel consumed by the system per hour, and Table 8 presents all values produced by the system. The results show that gaseous emissions polluting the environment increased due to the use of diesel fuel.

**Table 8:** The results using HOMER for diesel generator and PV panels.

Quantity	Value	Units
Diesel generator(Generic 25kW fixed capacity)	9.769	kWh/year
Diesel generator (Generic 25kW fixed capacity)	15,024.51	$
NAS Battery	1	Unit
NAS Battery	380,000.00	$
System inverter	600.00	$
Total fuel consumed	3.956	L
Average fuel per day	10.8	L/day
Average fuel per hour	0.452	L/hour
Carbon dioxide	10.357	Kg/year
Carbon monoxide	64.6	Kg/year
Unburned hydrocarbons	2.85	Kg/year
Particulate matter	0.388	Kg/year
Sulfur dioxide	25.4	Kg/year
Nitrogen oxides	60.8	Kg/year

A 2-kWh converter was used to convert power from DC to AC and vice versa as needed. Table 9 presents the parameters of operation hours and the converted energy of the converter.

**Table 9:** The Parameters and the energy converted/

Quantity	Inverter	Rectifier	Units
Capacity	2	2	kW
Mean output	0	0.357	kW
Hours of operation	0	1563	Hrs/year
Energy out	0	3126	kWh/year
Energy in	0	3291	kWh/year
Losses	0	165	kWh/year

### System with only a wind turbine

**Figure 12:**
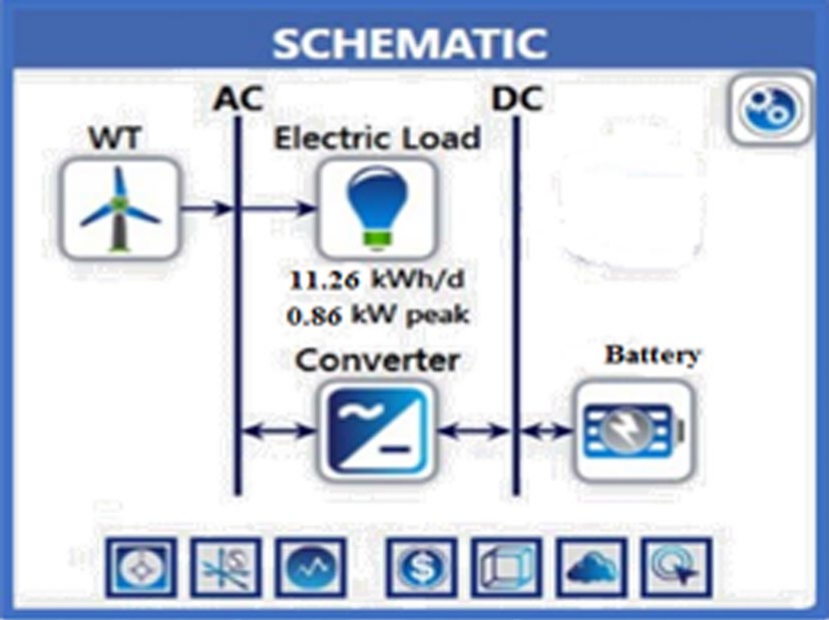
HOMER Schematic for wind turbine system.

In this model, a wind turbine was used as a renewable energy source. The system components include a generic 3 kW wind turbine, NAS battery, and rectifier system Fig. 12. This model does not use any fuel; thus, there are no emissions of polluting gases to the environment.

**Figure 13:**
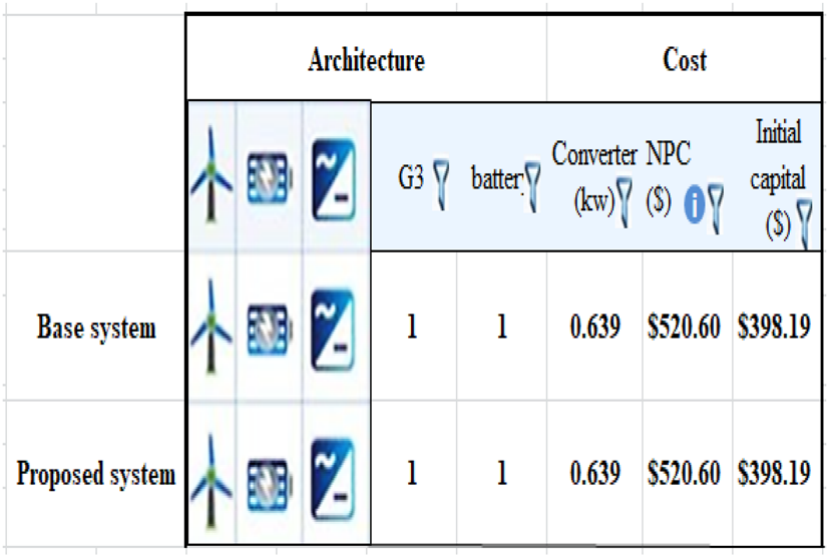
Screenshot for optimization results of wind turbine.

**Figure 14:**
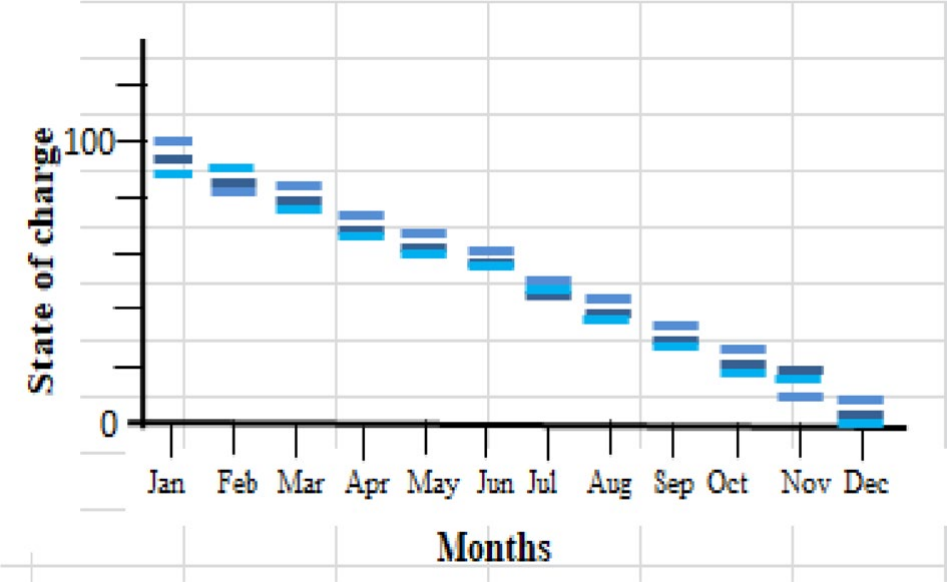
Screenshot for Monthly state of battery charging.

**Figure 15:**
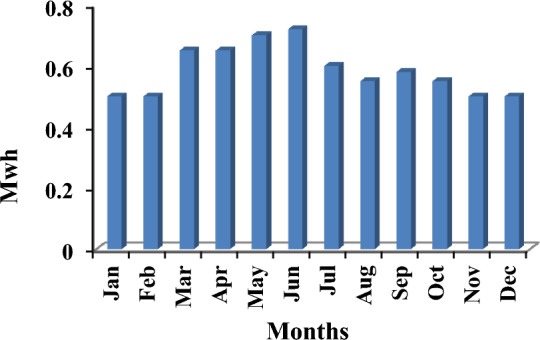
Screenshot for Monthly electrical power production of the system.

**Figure 16:**
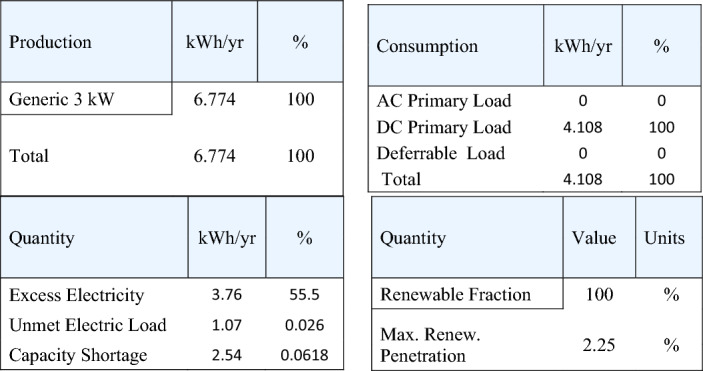
Screenshot for Power production & consumption at HOMER.

As previously mentioned, the electrical power that exceeds the load consumption needs to be stored using a battery. Figure 13 shows a screenshot of the optimization results of the wind turbine. Figure 14 displays the monthly state of battery charging. Figure 15 depicts the electrical power production of the system per month. A screenshot of the power production and consumption of HOMER is illustrated in Fig. 16. All the calculated results by the HOMER software are shown in Table 10.

**Table 10:** HOMER output results for wind turbine.

Quantity	Value	Units
System contents wind turbine (Generic 3 kW), NAS battery and system converter		
Operating cost	9468.79	$
Wind turbine (Generic 3 kW)	6.774	kWh/year
Battery	1	Unit
Battery losses	204	kWh/year
Rectifier hours of operations	7.374	Hrs/year
Losses of rectifier	151	kWh/year
Carbon dioxide	0	Kg/year
Carbon monoxide	0	Kg/year
Unburned hydrocarbons	0	Kg/year
Particulate matter	0	Kg/year
Sulfur dioxide	0	Kg/year
Nitrogen oxides	0	Kg/year

### On grid photovoltaic system

**Figure 17:**
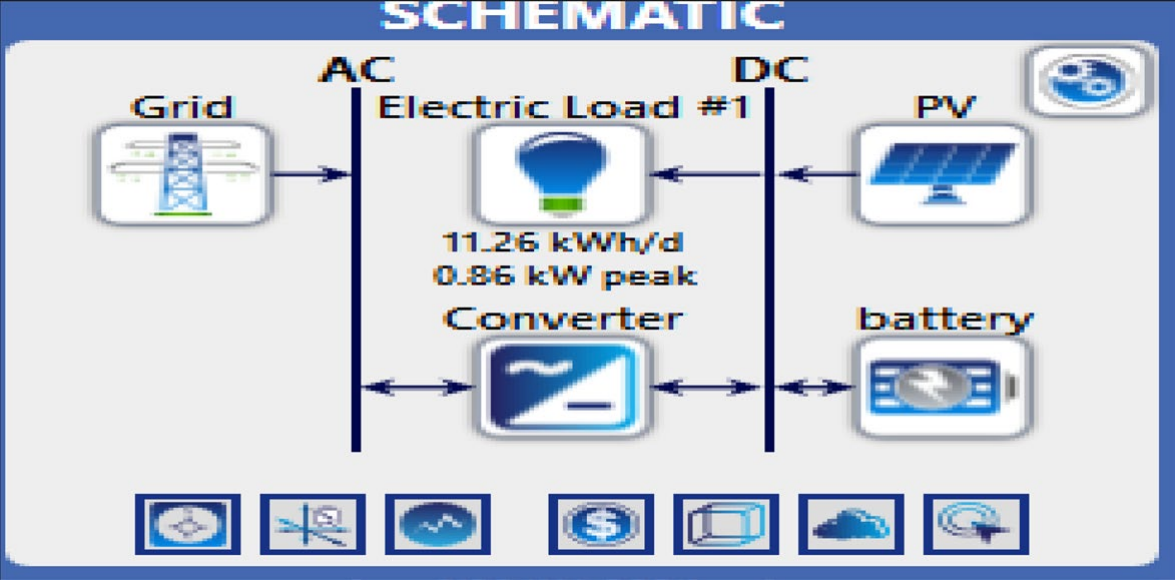
HOMER Schematic for Grid Connected with PV Model.

In this model, more than one source of electrical energy is studied, including a solar power station, which is a source of renewable electrical energy. The system is illustrated in Fig. 17, as well as the use of the electrical network, especially during downtime (whether at night or in the presence of clouds or rain). Moreover, a battery can be used to store solar energy if the system needs to be used at night. An inverter is also used in this system to convert power from AC to DC to feed loads or store electrical energy in the battery.

**Figure 18:**
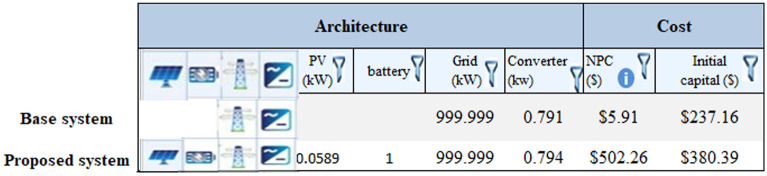
Screenshot for optimization results at ON-Grid model.

**Figure 19:**
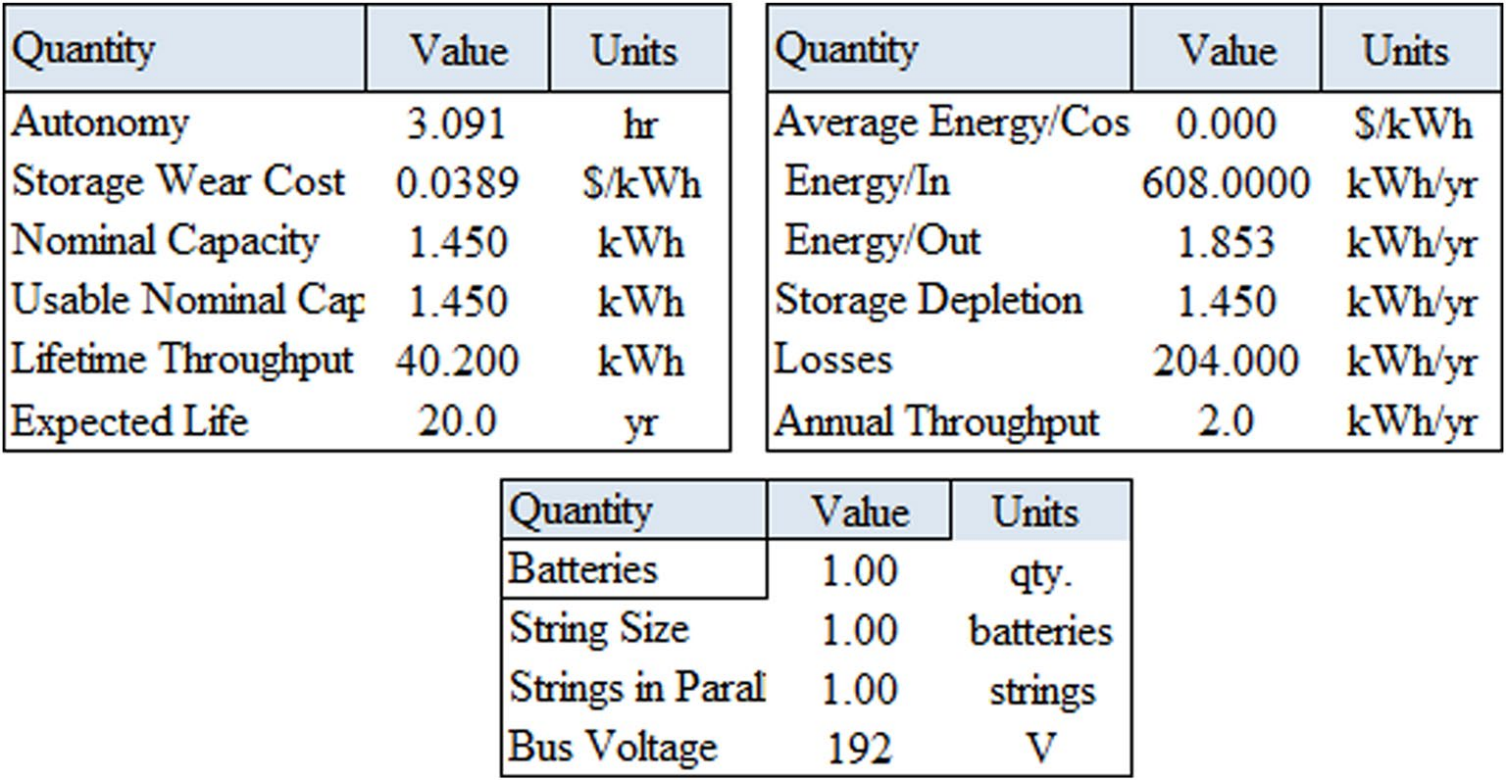
Screenshot for Power production and consumption and battery size at HMMER.

**Figure 20:**
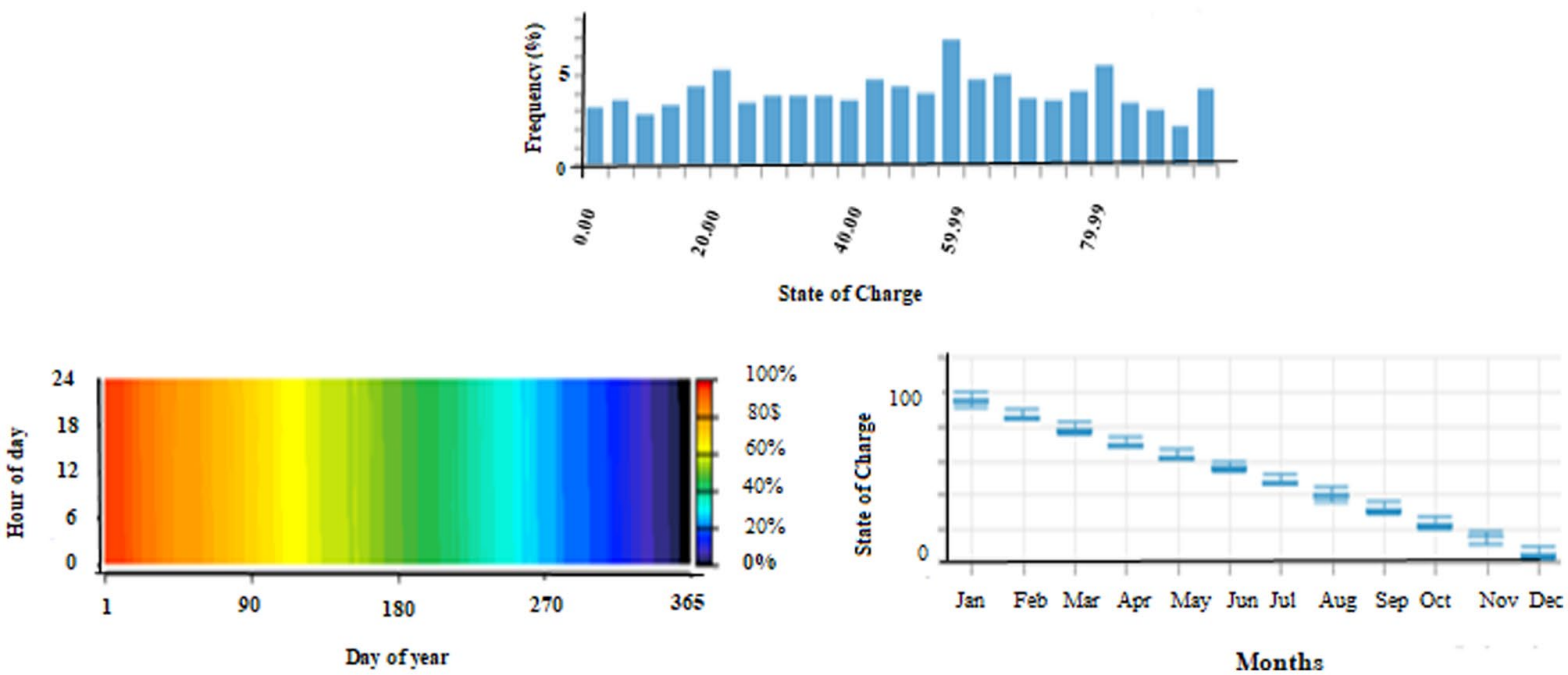
Screenshot for battery charging at HOMER.

Figure 18 shows a screenshot of the optimization results of the ON-Grid model, which contains one battery for storing power during the operational hours of the PV system. Further details regarding the electrical energy output and battery storage are presented in Fig. 19. While Fig. 20 shows a screenshot of the charging frequency and the monthly levels of battery charging. The output analysis of this model using HOMER is summarized in Table 11.

**Table 11:** HOMER output results for ON-Grid system with PV.

Quality	Value	Units
System contents		
Generic flat PV panel connected with the grid and using NAS battery and converter		
Generic flat PV total cost	154.72	$
NAS battery	4273.17	$
Battery lifetime	40	Years
Carbon dioxide	2349	Kg/year
Carbon monoxide	0	Kg/year
Unburned hydrocarbons	0	Kg/year
Particulate matter	10.2	Kg/year
Sulfur dioxide	0	Kg/year
Nitrogen oxides	0	Kg/year

**Figure 21:**
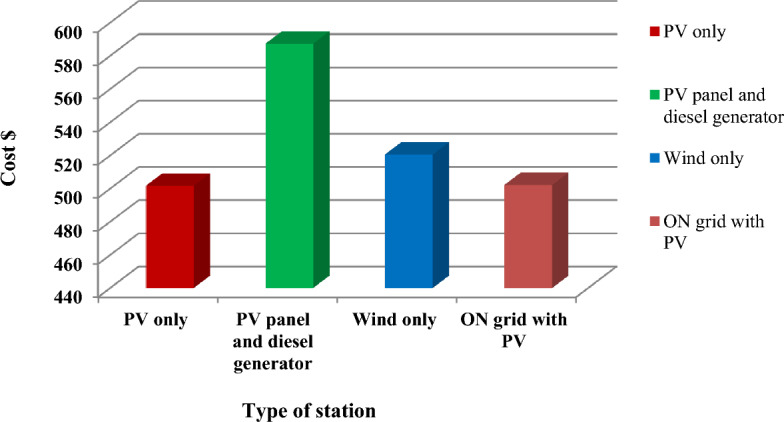
Total cost of all stations.

The results of each hybrid system used in this study in previous sections and presented them separately. The results are compared according to system cost and the presence of environmental emissions pollutants as presented in Table 12, while the comparison of all costs results is illustrated in Table 13. Furthermore Fig. 21 presents the comparison between total cost of all stations. Further comparisons were made between the results of this study with previous studies, which are illustrated in Appendix A.

**Table 12:** Environmental pollutant emissions for all systems.

	Carbon dioxide Kg/year	Carbon monoxide Kg/year	Unburned hydrocarbons Kg/year	Particulate matter Kg/year	Sulfur dioxide Kg/year	Nitrogen oxides Kg/year
PV only	0	0	0	0	0	0
PV panel and diesel generator	10.36	64.60	2.85	0.38	25.40	60.80
Wind turbine only	0	0	0	0	0	0
ON- Grid with PV system	2349	0	0	10.2	0	0

**Table 13:** Cost optimization analysis for all the system.

	System equipment	Capital cost ($)	Replacement cost ($)	O&M cost ($)	Fuel ($)	Salvage ($)	Total cost ($)	Total system cost
PV only	Generic flat plate PV	4.083	0	211.15	0	0	4294.54	
	NAS battery	380	121.15	64,637.99	0	68.273	497.510	
	Total system	384.08	121.15	64.848	0	68.273	501.804	501.804
PV panel and diesel generator	Generic 25 KW fixed capacity Gen. set	12.5	11,395.36	15,154.28	51,145.70	1182.82	89,012.55	
	NAS battery	380	121.15	64,637.99	0	68.273	497.510	
	System converter	600	254	0	0	47.91	806.65	
	Total System	393.1	132,791.86	51,145.7	69,504.72	69,504.72	587,329.59	587,329.59
Wind turbine only	Generic 3KW	18.0	5738.53	2326.95	0	53,234.03	22,831.45	
	NAS Battery	380.00	121,146.79	64,637.58	0	15.32	497,510.39	
	System converter	191.79	81.37	0	0	15.32	257.85	
	Total system	398,191.79	126,966.7	66,964.54	0	71,523.33	520,599.69	520.599.69
ON- Grid with PV system	Generic flat plate PV	147.22	0	7.61	0	0	154.83	
	Grid	0	0	4273.17	0	0	4273.17	
	NAS Battery	380.0	121,146.79	64,637.58	50	68,273.99	497,510.39	
	System converter	238.14	101.04	0	0	19.02	320.16	
	Total system	380.385	121,247.83	68,918.36	0	68,293.0	502,258.55	502,258.55

From the comparison of the results of the HOMER program for the costs and environmental polluting emissions for all systems, the optimal system is the system with only PV panels supplying the LTE mobile station with electrical energy. This study evaluated the conclusion of the best system to reduce polluting elements of the environment while maintaining the lowest possible cost compared to several previous studies mentioned in this study.

## Conclusion

This study compared four hybrid systems to produce the electricity needed to supply the LTE mobile phone station in 6th of October City, Egypt. The results were calculated and analyzed using the Homer program, which proved that the use of photovoltaic power plants is environmentally and economically optimal. This is consistent with the goal of the COP27 recommendations, which is to expand the use of sustainable energy while reducing the number of units used in the system, especially batteries, and thus obtaining an optimal design of the electrical system.

### Supplementary Information

Supplementary Tables.

## Data Availability

All data generated or analyzed during this study are included in this published article.

## References

[1] Hickmann T, Widerberg O, Lederer M, Pattberg P (2021). The United Nations Framework Convention on Climate Change Secretariat as an orchestrator in global climate policymaking. Int. Rev. Adm. Sci..

[2] Greene, L.A., 2000. EHPnet: United Nations Framework Convention on Climate Change.

[3] Breidenich C, Magraw D, Rowley A, Rubin JW (1998). The Kyoto protocol to the United Nations framework convention on climate change. Am. J. Int. Law.

[4] Abdel Gelil, I., 2018. History of Climate Change Negotiations and the Arab Countries The Case of Egypt. Climate Change and Environment in the Arab World.

[5] Jungudo, M.M., 2022. The impact of climate change in EGYPT. International Journal of Research (IJR), Vol.9.

[6] Bahgaat NK, Salam NA, Roshdy MM, Sakr SAE (2021). Design of solar system for LTE networks. Research Anthology on Clean Energy Management and Solutions.

[7] Akkucuk U (2015). Handbook of Research on Developing Sustainable Value in Economics, Finance, and Marketing.

[8] Ratheesh R, Vetrivelan P (2016). Power optimization techniques for next generation wireless networks. IACSIT Int. J. Eng. Technol..

[9] Chen T, Yang Y, Zhang H, Kim H, Horneman K (2011). Network energy saving technologies for green wireless access networks. IEEE Wirel. Commun..

[10] Chen Y, Zhang S, Xu S, Li GY (2011). Fundamental trade-offs on green wireless networks. IEEE Commun. Mag..

[11] Bogucka H, Conti A (2011). Degrees of freedom for energy savings in practical adaptive wireless systems. IEEE Commun. Mag..

[12] Boxwell M (2012). Solar Electricity Handbook: A Simple, Practical Guide to Solar Energy: How to Design and Install Photovoltaic Solar Electric Systems.

[13] Kumari M (2016). Use of Solar power in telecom tower to reduce environmental pollution. Int. J. Environ. Sci..

[14] Kosmopoulos P, Kazadzis S, El-Askary H. (2020). The Solar Atlas of Egypt. Geo-Cradle.

[15] Okba E, Zareef A, Badawy E (2021). Sustainable infrastructure assessments in remote areas in Egypt. HBRC J..

[16] OASYS South Asia Research Project. Off-grid Electricity Generation with Renewable Energy-Technologies in India: An Application of HOMER. Institute of Energy and Sustainable Development, De Montfort University-UK., 2019; doi: 10.1016/j.renene.2013.07.02.

[17] New & Renewable Energy Authority-Annual Report (2021). New & Renewable Energy Authority.

[18] Yaouba, Falama R, Welaji F, Soulouknga M, Mbakop F, Dadj´e A. Optimal Decision-Making on Hybrid Off-Grid Energy Systems for Rural and Remote Areas Electrification in the Northern Cameroon. Hindawi-J. Electr. Comput. Eng. Doi: 10.1155/2022/53/16520. (2022).

[19] United Nations, Transforming our World: Sustainable Development Plan 2030. UN., 2015.

[20] United Nations, Climate Action- What is renewable energy. https://www.un.org/en/climatechange. Accessed July 2022.

[21] Small Wind Guidebook. https://windexchange.energy.gov/small-wind-guidebook. Accessed July 2022.

[22] Small Wind Electric Systems-A U.S. Consumer’s Guide. U.S. Department of Energy -f Energy Efficiency and Renewable Energy. 2007.

[23] Bread A, Bread G, Alternative Energy Technology. Dar Al-Farouk, 2010 (Arabic Version 2018).

[24] Solar panel output explained. https://news.energysage.com/what-is-the-power-output-of-a-solar-panel/. Accessed July 2022.

[25] Burton T, Jenkins N, Sharpe D, Bossanyi E (2011). Wind Energy Handbook.

[26] Pulido D (2019). Energy Storage Technologies for Off-grid Houses.

[27] Pulido Q (2017). Practical approach in glycerol oxidation for the development of a glycerol fuel cell. Trends Green Chem..

[28] Abdel-Rahman A, Ali A, Abdel-Rady A, Ookawara S, An Analysis of Thermal Comfort and Energy Consumption within Public Primary Schools in Egypt. The Asian Conference on Sustainability, Energy and the Environment. Egypt, 2014.

[29] Salah SI, Eltaweel M, Abeykoon C (2022). Towards a sustainable energy future for Egypt: A systematic review of renewable energy sources, technologies, challenges, and recommendations. Clean. Eng. Technol..

[30] The Solar Atlas of Egypt URL: http://www.nrea.gov.eg/Content/files/SOLAR%20ATLAS%202018%20digital1.pdf.

[31] New & Renewable Energy Authority-Annual Report 2020. New & Renewable Energy Authority, 2020.

[32] Ministry of Electricity and Energy New and Renewable Energy Authority (NERA), Feasibility Study for a Large Wind Farm at Gulf of El Zayat. 2008.

[33] Lamayer Alliance International LLC and Ecoda Environmental Consulting Company, Strategic and Cumulative Environmental and Social Assessment - Program for Effective Wind Turbine Management for Wind Energy Projects in the Gulf of Suez, 2018.

[34] Mclaughlin S, Grant PM, Thompson JS, Haas H, Laurenson DI, Khirallah C, Wang R (2011). Techniques for improving cellular radio base station energy efficiency. IEEE Wirel. Commun..

[35] Jahid A, Islam MS, Hossain MS, Hossain ME, Monju MKH, Hossain MF (2019). Toward energy efficiency aware renewable energy management in green cellular network with joint coordination. IEEE Access.

[36] Alsharif MH, Nordin R, Ismail M (2015). Energy optimisation of hybrid off-grid system for remote telecommunication base station deployment in Malaysia. J. Wirel. Commun. Netw..

[37] Huawei. Mobile Networks Go Green. http://www.huawei.com/en/abouthuawei/publications/communicaite/hw-082734.htm. Accessed 10 October 2020.

[38] Hossain MS, Ziaul Islam K, Jahid A, Rahman KM, Ahmed S, Alsharif MH (2020). Renewable energy-aware sustainable cellular networks with load balancing and energy-sharing technique. Sustainability.

[39] Hossain MS, Rahman MF (2020). Hybrid solar PV/Biomass powered energy efficient remote cellular base stations. Int. J. Renew. Energy Res. (IJRER).

[40] Jahid, A.; Hossain, M.S. Energy-cost aware hybrid power system for off-grid base stations under green cellular networks. In Proceedings of the IEEE International Conference on Electrical Information and Communication Technology (EICT), Khulna, Bangladesh, 7–9 December 2017; pp. 1–6.

[41] Jahid A, Monju MKH, Hossain ME, Hossain MF (2018). Renewable energy assisted cost aware sustainable off-grid base stations with energy cooperation. IEEE Access.

[42] Hossain, M.S.; Jahid, A.; Rahman, M.F. Quantifying potential of hybrid PV/WT power supplies for off-grid LTE base station. In Proceedings of the IEEE International Conference on Computer, Communication.

[43] Sustainable Energy Use in Mobile Communications, Ericsson Inc., White Paper. 2007. https://www.techonline.com/electrical-engineers/education-training/tech-papers/4136182/Sustainable-Energy-Use-in-Mobile-Communications. Accessed 10 October 2020.

[44] E-plus, Nokia Siemens Networks Build Germany First Offgrid Base Station. 2011. http://www.nokiasiemensnetworks.com. Accessed 10 October 2020.

[45] Akkucuk U, Akkucuk U (2016). SCOR model and the green supply chain. Handbook of Research on Waste Management Techniques for Sustainability.

[46] Krawczeniuk, N., 2019. Analysis of LTE network RF performance in a dense urban environment.

[47] Ike D, Adoghe AU, Abdulkareem A (2014). Analysis of telecom bas stations powered by solar energy. Int. J. Sci. Technol. Res..

[48] Alsharif MH (2017). Comparative analysis of solar-powered base stations for green mobile networks. Energies.

[49] Alsharif MH, Kim J, Kim JH (2017). Green and sustainable cellular base stations: An overview and future research directions. Energies.

[50] Barnes DF (2019). Electric Power for Rural Growth: How Electricity Affects Rural Life in Developing Countries.

[51] Thomsen N, Wagner T, Hoisington A, Schuldt S (2019). A sustainable prototype for renewable energy: Optimized prime-power generator solar array replacement. Int. J. Energy Prod. Manag..

[52] Mostafa E, Bahgaat NK (2017). A comparison between using a firefly algorithmand a modified pso technique for stability analysis of a pv system connected to grid. Int. J. Smart Grid.

[53] Mostafa EEA, Bahgaat NK, El Sayed ME, Othman E-SA (2017). Voltage stability for a photovoltaic system connected to grid by using genetic algorithm technique. Int. J. Grid Distrib. Comput..

[54] Kumar PP, Saini RP (2020). Optimization of an off-grid integrated hybrid renewable energy system with different battery technologies for rural electrification in India. J. Energy Storage.

[55] Yıldırım IK, Baysal U, Yıldırım IK, Baysal U (2019). Comparison of MPPT and PWM methods on designing microcontroller based power control unit for wireless sensor networks. 2019 6th International Conference on Electrical and Electronics Engineering (ICEEE).

[56] Zhang Y, Yan S, Yin W, Wu C, Ye J, Wu Y, Liu L (2023). HOMER-based multi-scenario collaborative planning for grid-connected PV-storage microgrids with electric vehicles. Processes.

[57] Han T, Ansari N (2014). Powering mobile networks with green energy. IEEE Wirel. Commun..

[58] Ibrahim MH, Ibrahim MA (2021). Solar-wind hybrid power system analysis using homer for Duhok, Iraq. Prz. Elektrotechniczny.

[59] Thirunavukkarasu M, Sawle Y (2020). Design, analysis and optimal sizing of standalone PV/diesel/battery hybrid energy system using HOMER. IOP Conf. Ser. Mater. Sci. Eng..

[60] Fantidis J, Bandekas D, Vordos N (2015). Study of a wind/PV/battery hybrid system—Case study at Plaka in Greece. J. Eng. Sci. Technol. Rev..

[61] Boxwell M (2010). Solar Electricity Handbook: A Simple, Practical Guide to Solar Energy-Designing and Installing Photovoltaic Solar Electric Systems.

[62] Mina R, Sakr G (2019). Design and optimization of a renewable-energy fully-hybrid power supply system in mobile radio access networks. Int. J. Renew. Energy Res. (IJRER).

[63] Chiaraviglio L, Amorosi L, Blefari-Melazzi N, Dell’Olmo P, Mastro AL, Natalino C, Monti P (2019). Minimum cost design of cellular networks in rural areas with UAVs, optical rings, solar panels, and batteries. IEEE Trans. Green Commun. Netw..

[64] Kumar, P. P. & Saini, R. P. Optimization of an off-grid integrated hybrid renewable energy system with various energy storage technologies using different dispatch strategies. *Energy Sour. A Recov. Util. Environ. Effects* 1–30 (2020).

[65] NGK Insulators, LTD. Sodium-Sulfur (NAS) Batteries https://www.ngk-insulators.com/en/product/nas.html.

