# Supplementary material for: Estimation of renewable energy systems for mobile network based on real measurements using HOMER software in Egypt
Source: Sci Rep. 2023 Oct 4;13:16713. doi: 10.1038/s41598-023-43877-2 (PMC10550915; doi:10.1038/s41598-023-43877-2)
Supplement: Supplementary file 1 — Supplementary Tables. [file 41598_2023_43877_MOESM1_ESM.docx]

**Appendix A**

Table A1. The technical and cost values of the batteries

|  | Previous study | | | This study |
| --- | --- | --- | --- | --- |
| Battery type | Lead Acid (PbSO4) [55] | Lithium Iron Phosphate (LiFePO4) [55] | Nickel Iron (Ni-Fe) [55] | BASF NAS lithium-ion [6, 65] |
| Manufacturer | Trojan [55] | Victron [55] | Iron Edison [55] | NGK instruments [65] |
| Model | SSIG 06 490 | LFP-12.8/200-a | TN 1000 | BASF- NAS |
| Nominal capacity (SBAT) | 490 A | 300 A | 1000A | 1200 A |
| Nominal voltage (VBAT) | 6 v | 12.8 v | 1.2 v | 2 V  192 v ( path voltage) |
| Operating temperature | −20 °C to +45 °C | −20 °C to +50 °C | -30⁰ C to +60⁰ C | -20 to +45 |
| Capital cost (CC) | 410 $ | 3317 $ | 1057 $ | 380 $ |
| Annual O&M cost | 2.5% of CC | No maintenance | 2% of CC | 64.637 $ |
| Battery design life | 3 years @70% *DOD  2.5 years @80% DOD | 15 years @50% DOD  9 years @70% DOD  7.5 years @80% DOD | 30 years+ | 20 year |

*DOD = Depth of Discharge

Table A2. Comparison between the system components used in the study and previous studies:

|  |  | Previous studies | Reference Number | This study |
| --- | --- | --- | --- | --- |
| 1 | Battery type | Lead Acid (LA), Lithium-Ion (Li-Ion), and Nickel-Iron (Ni-Fe) | [55, 56] | BASF NAS lithium-ion |
|  | Number of batteries used | 51 | [6] | 1 string [64] |
|  | Cost of PV | 324200 EGP= 19007 $ (in 2020) | [6] | 4.294.54 $ ( in 2023) |
|  | Cost of battery | 255.000 EGP = 15.000 $ ( in 2020 ) | [6] | 497.510 $ ( in 2023) |
|  | Battery capacity | 91.800 KWh | [6] | 1250 kWh  long duration (4.4 h) |
|  | Battery life time | 10 year | [6] | 20 y or 6,250,000 kWh |
|  | amount of energy needed | Daly = 60.35 kWh  528.666 Kwh/year ( PV only) | [6] | 2976 Kwh/year (PV only ) |
|  | Operation cost | 3242 $ | [6] | 501.804 $ |

It should be taken into account that it is not possible to rely on the current price for comparison between previous studies and this study due to the difference in the currency price. However; it is also important to consider the number of units used in electrical design. For example, the number of batteries and the number of solar energy units as a good means of comparison, given that the fewer the number of units. The actual cost is reduced.

Furthermore; taking into justification the different prices of the components according to each of their types and the producing companies as well, but these prices can be considered indicative only at the time of conducting this study.

Table A3. On-Grid PV system components and cost

|  | Pv Kw/h | Grid KW | Converter Kw | Total power production KW/year | COE $ | NPC $ |
| --- | --- | --- | --- | --- | --- | --- |
| Previous study [66] | 64,403 | 8 | 21 | 72.909 | 0.525 | 429.326 |
| This study | 0.598 | 1 | 0.794 | 107 | 7.88 | 502.258 |
